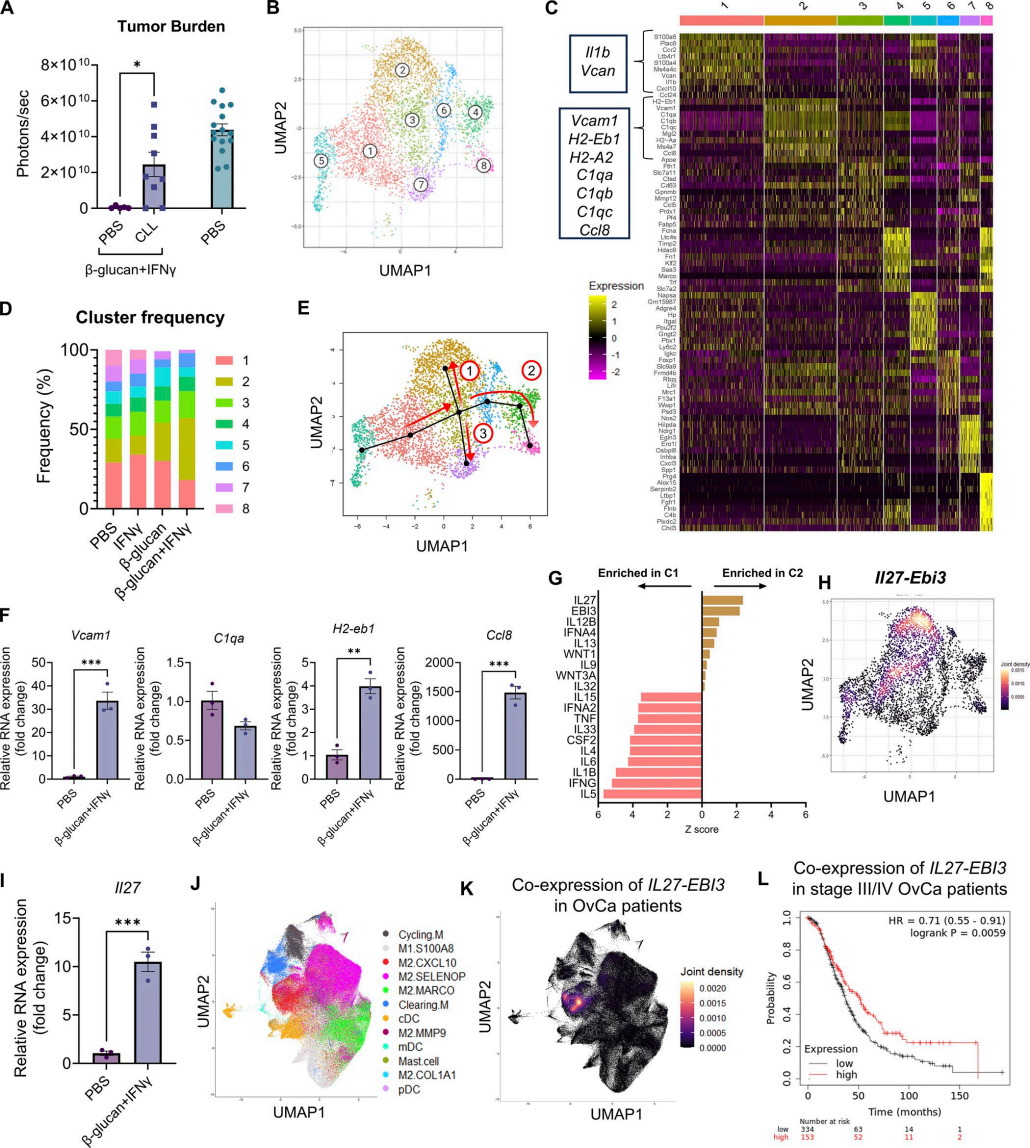# Correction: Myeloid activation clears ascites and reveals IL27-dependent regression of metastatic ovarian cancer

**DOI:** 10.1084/jem.2023196702272025c

**Published:** 2025-03-11

**Authors:** Brennah Murphy, Taito Miyamoto, Bryan S. Manning, Gauri Mirji, Alessio Ugolini, Toshitha Kannan, Kohei Hamada, Yanfang P. Zhu, Daniel T. Claiborne, Lu Huang, Rugang Zhang, Yulia Nefedova, Andrew Kossenkov, Filippo Veglia, Rahul Shinde, Nan Zhang

Vol. 221, No. 12 | https://doi.org/10.1084/jem.20231967| November 21, 2024

The authors regret that their original article contained typographical errors. The unit of measure for recombinant mouse IFNγ has been corrected in the Materials and methods section. The corrected paragraph, with the revised text in bold, is shown here.

## Tumor implantation, treatment, and survival

Cells were harvested with trypsin-EDTA (corning), washed in PBS, and injected i.p. into mice. 3 × 10^6^ ID8 cells were injected in 100 μl PBS into 8–10-wk-old female WT mice and allowed to seed for 2 wk prior to β-glucan treatment. Mice were treated with 500 µg soni- cated whole β-glucan particles (tlrl-wgp; Invivogen) in PBS i.p. or PBS vehicle control once every other week for 2 wk for a total of two injections (Fig. S1 A). 2 wk after the final dose of β-glucan, tumor burden was assessed by IVIS Spectrum Imaging (PerkinElmer). For KPCA tumors, 1 × 10^6^ KCPA cells were injected i.p. into WT or IFNγR KO in 200 μl of a 1:1 matrigel:PBS mix (Matrigel Matrix Basement Membrane, 354234; Corning). Tumors grew for 1 wk prior to treatment. **Mice were treated with 500 µg β-glucan, 20 µg recombi- nant mouse IFNγ (315-05; Peprotech), βI, or PBS vehicle control once a week for 2 wk (Fig. S1 B).** 1 wk after the final treatment, mice were imaged by IVIS. Importantly, β-glucan was sonicated intermittently on high for 15 min immediately prior to injection to ensure thorough disruption of β-glucan aggregates. Recombinant mouse IFNγ was gently reconstituted in molecular grade, sterile H_2_O and diluted in PBS to its working concentration. To preserve IFNγ activity, solutions were handled gently to reduce the presence of bubbles and never vortexed. For macrophage depletion studies, mice were treated with 100 μl of CLL (C-005; Liposoma) 1 wk prior to cancer capture studies. For tumor studies, 100 μl of CLL was injected i.p. 5, 9, 14, and 19 days after tumor seeding. On day 14, CLL was administered 4 h before BI treatment. For T cell depletion studies, αCD4 (C2838; Leinco Tech) and αCD8 (C2850; Leinco Tech) monoclonal antibodies were injected 150 μg each i.p. in WT KPCA tumor-bearing mice 3 days following cancer seeding and then once a week for 2 wk for a total of three injections. For the IL27 neutralization studies, 200 µg αIL27p28 monoclonal antibody (BE0326; InVivoMAb) (Marillier et al., 2014) was injected 2 days prior to βI treatment, at the same time as treatment, and then two times a week for 2 wk following treatment for a total of six injections. For treatment of experiments with carboplatin, 10–30 mg/kg once a week of carboplatin was administered with or without βI, starting at day 7 for 2 wk. The dose and timing of β-glucan and IFNγ were the same as mentioned above. On day 21, mice were imaged by IVIS and further monitored for survival analysis.

In addition, the genes *H2-A2* and *C1qa* in Fig. 4 C should have been listed on separate lines. The revised figure is shown here. The errors appear in print and in PDFs downloaded before February 28, 2025.

**Figure:**